# Current status of and future prospects for the treatment of unresectable or metastatic gastrointestinal stromal tumours

**DOI:** 10.1007/s10120-023-01381-6

**Published:** 2023-03-13

**Authors:** Yoichi Naito, Toshirou Nishida, Toshihiko Doi

**Affiliations:** 1 Department of General Internal Medicine, National Cancer Center Hospital East, 6-5-1 Kashiwanoha, Kashiwa, Chiba 277-8577 Japan; 2 Department of Experimental Therapeutics, National Cancer Center Hospital East, Kashiwa, Japan; 3 Department of Medical Oncology, National Cancer Center Hospital East, Kashiwa, Japan; 4 Department of Surgery, Japan Community Health Care Organization Osaka Hospital, Osaka, Japan; 5 National Cancer Center Hospital, Tsukiji, Tokyo, Japan

**Keywords:** Fourth-line treatment, Gastrointestinal stromal tumours, Refractory GIST

## Abstract

Gastrointestinal stromal tumours (GISTs) are soft-tissue sarcomas of the gastrointestinal tract. Surgery is the standard treatment for localised disease, but the risk of relapse and progression to more advanced disease is substantial. Following the discovery of the molecular mechanisms underlying GISTs, targeted therapies for advanced GIST were developed, with the first being the tyrosine kinase inhibitor (TKI) imatinib. Imatinib is recommended in international guidelines as first-line therapy to reduce the risk of GIST relapse in high-risk patients, and for locally advanced, inoperable and metastatic disease. Unfortunately, imatinib resistance frequently occurs and, therefore, second-line (sunitinib) and third-line (regorafenib) TKIs have been developed. Treatment options are limited for patients with GIST that has progressed despite these therapies. A number of other TKIs for advanced/metastatic GIST have been approved in some countries. Ripretinib is approved as fourth-line treatment of GIST and avapritinib is approved for GIST harbouring specific genetic mutations, while larotrectinib and entrectinib are approved for solid tumours (including GIST) with specific genetic mutations. In Japan, pimitespib, a heat shock protein 90 (HSP90) inhibitor, is now available as a fourth-line therapy for GIST. Clinical studies of pimitespib have indicated that it has good efficacy and tolerability, importantly not displaying the ocular toxicity of previously developed HSP90 inhibitors. Additional approaches for advanced GIST have been investigated, including alternative uses of currently available TKIs (such as combination therapy), novel TKIs, antibody–drug conjugates, and immunotherapies. Given the poor prognosis of advanced GIST, the development of new therapies remains an important goal.

## Gastrointestinal stromal tumours (GIST)

### Epidemiology

Gastrointestinal stromal tumours (GISTs) are the most frequent soft-tissue sarcoma of the gastrointestinal (GI) tract. They are usually found in the stomach (60–65% of cases) and small intestine (20–35% of cases), with the rectum, colon and oesophagus more rarely involved [1]. GISTs usually develop from precursors of the interstitial cells of Cajal found in the myenteric plexus, which are responsible for maintaining and controlling GI motility [1]. Not all GISTs show malignant behaviour, but 20–25% of gastric and 40–50% of small intestinal GISTs do show clinically malignant behaviour [2].

Population-based studies from various European countries and data from the United States (US) National Cancer Institute estimate the incidence of GISTs (clinical, symptomatic or requiring treatment) to be approximately 6–22 cases per million per year [3]. GISTs are slightly more common in males than females, and are predominantly a disease of older age, with a median age at diagnosis of approximately 60–65 years [4].

### Molecular oncology

A major advance in the treatment of GISTs came with the discovery of the molecular mechanisms involved in their oncogenesis, most frequently gain-of-function mutations in v-kit Hardy-Zuckerman 4 feline sarcoma viral oncogene homolog (*KIT*) [5] or platelet-derived growth factor receptor alpha (*PDGRFA*) [6, 7] genes. Approximately 60–80% of GISTs have a *KIT* mutation, and approximately 5–15% have a *PDGRFA* mutation [1, 8], which result in ligand-independent activation of kinases, believed to be the main driver of GIST development and maintenance. GISTs lacking mutations of *KIT* or *PDGRFA* are termed ‘wild-type GISTs’, and are less frequently found [8]. Wild-type GISTs may have mutations in genes for v-raf murine sarcoma viral oncogene homolog B1 (BRAF), neurofibromin (NF), neurotrophic tyrosine receptor kinase (NTRK) and succinate dehydrogenase (SDH) [1, 8].

In this narrative review, we discuss the current status of and future prospects for the treatment of GIST, highlighting the differences between the management of this cancer type in Japan versus other countries.

## Current status of treatment for unresectable and advanced GIST

In patients with GIST, surgery is the standard treatment for localised disease, but the risk of relapse and progression to more advanced disease is substantial [4, 9, 10]. Unresectable and advanced/metastatic GISTs are resistant to standard cytotoxic chemotherapy and radiotherapy and, for many decades, the prognosis for patients with this stage of disease was poor, with a 5-year survival rate of approximately 12% [11], and median survival of 18–24 months [12]. However, knowledge of the mechanisms of oncogenesis has led to the development of targeted therapies for GISTs. This culminated in the early 2000s in the approval of the tyrosine kinase inhibitor (TKI) imatinib for the treatment of metastatic and/or unresectable malignant GISTs.

Imatinib, an inhibitor of KIT/PDGFRA tyrosine kinase, is now approved in many countries worldwide, including the US, Europe and Japan, and is recommended by international guidelines, including those of the Japan Society of Clinical Oncology, as first-line therapy for GISTs to reduce the risk of relapse in high-risk patients, and for locally advanced, inoperable/unresectable and metastatic disease [9, 10, 13–17]. However, some patients have GISTs that do not show any response to imatinib (primary resistance), and most GISTs eventually develop secondary resistance (defined as resistance occurring after > 6 months of clinical response) to imatinib. *KIT* exon 9 duplications confer limited response to imatinib and the requirement for higher doses [18, 19], while *PDGFRA* D842V substitution causes primary resistance to the drug [20]. Secondary imatinib resistance results from the development of newly acquired mutations in *KIT* or *PDGFRA* [21–23], which allows reactivation of tyrosine kinase. There is evidence to indicate that the susceptibility of imatinib-resistant GISTs to subsequent treatments is dependent on the site of the acquired *KIT* mutation [24].

The US and European guidelines suggest consideration of surgical resection in selected patients who have experienced a response to imatinib, despite the limited evidence for the benefit of such an approach [13–15]. A multicentre, randomised, controlled trial compared surgery to treat residual disease plus imatinib (n = 19) with imatinib alone (n = 22) in patients with recurrent/metastatic GIST that had responded to imatinib [25]. In this trial, no statistically significant difference was observed between the two treatment regimens in 2-year progression-free survival (PFS), although overall survival (OS) was significantly longer in the surgery plus imatinib group [25]. However, the usefulness of this approach is currently unclear (given the small sample size in the aforementioned study) and requires further investigation.

**Fig. 1:**
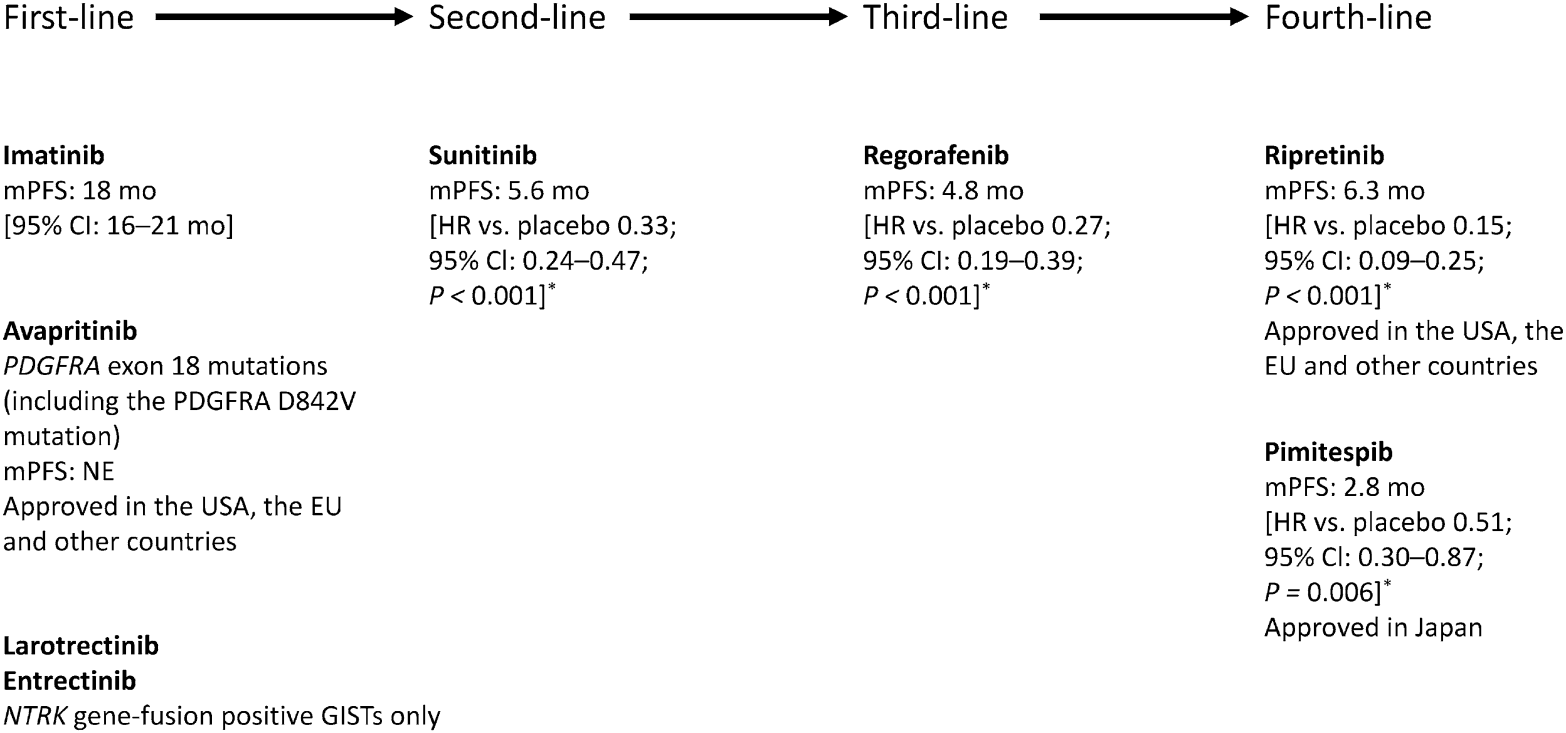
Agents for the treatment of advanced or metastatic gastrointestinal stromal tumours (GIST), based on the US National Comprehensive Cancer Network^®^ (NCCN^®^) [15] and Japan Society of Clinical Oncology [17] guidelines for the management of GIST. Median PFS is shown for imatinib [106], sunitinib [107], regorafenib [32], ripretinib [35], avapritinib [108], and pimitespib [71]. Adapted with permission from the NCCN Clinical Practice Guidelines in Oncology (NCCN Guidelines^®^) for Gastrointestinal stromal tumors (GISTs) Version 1 2022^©^. 2022 National Comprehensive Cancer Network, Inc. All rights reserved. The NCCN Guidelines^®^ and illustrations herein may not be reproduced in any form for any purpose without the express written permission of NCCN. To view the most recent and complete version of the NCCN Guidelines, go online to NCCN.org. The NCCN Guidelines are a work in progress that may be refined as often as new significant data becomes available. NCCN makes no warranties of any kind whatsoever regarding their content, use or application and disclaims any responsibility for their application or use in any way. *CI* confidence interval, *EU* European Union, *HR* hazard ratio, *mo* months, *mPFS* median progression-free survival, *NE* not estimable, *NTRK* neurotrophic tyrosine receptor kinase, *PDGRFA* platelet-derived growth factor receptor alpha, *USA* United States of America. *P value vs. placebo

To address the need for therapies for imatinib-refractory GIST, other TKIs have been approved (see Fig. 1). Sunitinib and regorafenib are now available in multiple countries around the globe, including Japan [26]. International guidelines recommended sunitinib as second-line and regorafenib as third-line treatment for GISTs [9, 10, 14–16, 27].

As mentioned above, most patients with GIST have mutations in *KIT* or *PDGFRA*, but some have wild-type GIST. For these latter patients, similar to other cancer treatments and as described in the US and European guidelines for GIST [14, 15], next-generation sequencing can be used to guide the treatment of those with *BRAF* and *NF1* mutations and fibroblast growth factor receptor (*FGFR*) fusions. It is anticipated that individualized genomic therapy will eventually become standard for the treatment of GIST.

Larotrectinib [28] and entrectinib [29] have received approval in the US for the treatment of any solid tumour harbouring neurotrophic tyrosine kinase (*NTRK*) fusions that are either metastatic or where surgical resection is likely to result in severe morbidity, and where there are no satisfactory alternative treatments or the cancer has progressed despite treatment. This indication can include advanced/refractory GIST. Larotrectinib and entrectinib are also approved for use in patients with *NTRK* fusion-positive advanced solid tumours in the European Union (EU), Japan and several other countries [30, 31].

Despite the availability of these treatments, options remain limited for patients with GISTs who do not respond to third-line therapy. The median PFS with placebo was only 0.9 months in the pivotal trial of third-line regorafenib therapy [32], indicating the poor prognosis for patients with refractory disease.

## Latest advances in the treatment of unresectable and advanced GIST

### International perspective

In May 2020, ripretinib was approved in the US for the treatment of GISTs in patients who had received prior treatment with ≥ 3 kinase inhibitors, including imatinib [33]. Ripretinib is a type II “switch-control” kinase inhibitor that inactivates the activation loop, via both antagonism (preventing the ‘switch’ from adopting a type I active state) and agonism (stabilising the type II inactive state) [34]. It is thought to inhibit all primary and secondary drug-resistant mutants of *KIT* and *PDGFRA* [34]. The approval of ripretinib was based on the phase 3 INVICTUS trial (n = 129), in which ripretinib improved median PFS relative to placebo (6.3 vs. 1.0 months, respectively), as well as objective response (9.5% vs. 0%, respectively) and OS (15.1 vs. 6.6 months, respectively); median duration of response (DOR) had not been reached at data cut-off [35]. Ripretinib has since been approved in Australia, Canada, China, the EU, Hong Kong, Switzerland, Taiwan, and the United Kingdom (UK) [36, 37]. Further, it is a fourth-line option for GISTs in the US National Comprehensive Cancer Network (NCCN) Clinical Practice Guidelines in Oncology (NCCN Guidelines^®^) [15, 27] and the European Society for Medical Oncology/European Reference Network for Rare Adult Solid Cancers/European Reference Network for Genetic Tumour Risk Syndromes Clinical Practice Guidelines [14]. Avapritinib was approved in the US in January 2020 for adults with unresectable or metastatic GIST harbouring a *PDGFRA* exon 18 mutation, including D842V mutations [38]. Avapritinib is a type I inhibitor of *KIT* and *PDGFRA* activation loop mutants, such as those of *PDGFRA* D842V and *KIT* exons 11 and 17 [39]. The efficacy of the drug was established in the phase 1 NAVIGATOR trial (n = 43; n = 38 with D842V mutations). At the time of approval, this trial found an overall response rate (ORR) of 84% at a median follow-up of 10.6 months, with 7% complete response (CR) and 77% partial response (PR) [38]. The median DOR, PFS and OS were not reached [38]. Among patients with *PDGFRA* D842V mutations, the ORR was 89%, CR was 8% and PR was 82%; the median DOR was not reached [38]. An updated analysis of NAVIGATOR reported that in patients with the D842V mutation, the ORR was 91%, clinical benefit rate (CBR) was 98%, median DOR was 27.6 months and median PFS was 34.0 months [40]. Median OS was not reached. The phase 3 VOYAGER trial found no significant difference in median PFS between avapritinib (n = 240) and regorafenib (n = 236) as third-line or later treatment in patients with unresectable or metastatic GIST [41]. Median PFS was 4.2 months with avapritinib versus 5.6 months with regorafenib. ORR was 17.1% and 7.2% for avapritinib and regorafenib, respectively, with median DORs of 7.6 and 9.4 months, respectively. Disease control rates (DCRs) were 41.7% and 46.2%, respectively. Avapritinib is now approved in several other countries, including China, Taiwan, Hong Kong and the EU [42]. In the EU, avapritinib monotherapy is indicated for the treatment of adult patients with unresectable or metastatic GIST harbouring the *PDGFRA* exon 18 mutation, including *PDGFRA* D842V mutations [43].

### Japanese perspective

There are options available in Japan for the treatment of patients with GISTs that are resistant to first- and second-line therapies. However, unlike the situation in several other countries, no agent had been specifically approved for fourth-line treatment (larotrectinib and entrectinib are approved for the more general indication of *NTRK* fusion-positive advanced solid tumours [44, 45]). As a result, other approaches to the management of patients with advanced or metastatic GIST are frequently used.

#### Non-pharmacological treatments

Non-pharmacological treatments include radiotherapy for metastatic lesions (although current guidelines do not recommend radiotherapy as a treatment for GIST because this type of cancer has traditionally been considered to be radiotherapy resistant, there have been some encouraging results in case reports/series [46, 47]), and hepatic artery embolisation [48, 49] and radiofrequency ablation [50] for liver lesions.

#### Rechallenge with TKIs

The reintroduction of a TKI that had previously been well tolerated and effective has proven beneficial in several studies. A retrospective, non-randomised study suggested that imatinib rechallenge combined with best supportive care (BSC) may have advantages over BSC alone in Japanese patients with locally advanced or metastatic GIST who had previously received both imatinib and sunitinib [51]. After a median follow-up of 7.2 months, median OS was 22 months for imatinib plus BSC (n = 14) compared with 4 months for BSC alone (n = 12) [*P* = 0.058]. There were three responders in the imatinib plus BSC group and one in the BSC alone group.

Encouraging effects have also been seen with TKI rechallenge as fourth-line treatment. The phase 3 Rechallenge of Imatinib in GIST Having no effective Treatment (RIGHT) study assessed the efficacy and safety of imatinib (n = 41) versus placebo (n = 40) in patients with metastatic and/or unresectable GIST who had experienced initial benefit from imatinib but had subsequently evolved to progression of GIST despite receiving treatment with at least imatinib and sunitinib (40% of patients had received ≥ 3 prior TKIs) [52]. After a median follow-up period of 5.2 months, median PFS was 1.8 months with imatinib compared with 0.9 months with placebo. Imatinib was associated with a 54% reduction in the risk of disease progression or death (*P* = 0.005).

A larger, but uncontrolled, Italian real-world study investigated the effects of imatinib rechallenge in 71 patients with advanced GIST whose disease had progressed after treatment with imatinib, sunitinib and regorafenib [53]. The median follow-up was 13 months; median time to progression was 5.4 months and OS was 10.6 months.

#### Pimitespib

Heat shock proteins (HSPs) are a class of adenosine triphosphate (ATP)-dependent proteins that act as molecular ‘chaperones’. They assist in a number of events that are essential for normal cellular functioning and survival, such as the folding of ‘client’ proteins [54]. The levels of HSPs increase in response to cellular stressors, including a rise in temperature, hence their name. The expression of HSPs is increased in tumour cells [55], helping to drive oncogenesis and tumour cell homeostasis and survival [54]. Many of the client proteins of HSP90 are signal transducers that are involved in cellular growth and control, and have been identified as cancer-related proteins necessary for tumour development, including KIT, PDGFRA, BRAF, vascular endothelial growth factor (VEGFR) and hypoxia-inducible factor 1 [54, 56]. HSP90 inhibition, therefore, represents a rational approach to the treatment of GIST.

A number of HSP90 inhibitors have been developed [57] and investigated as potential treatments for GIST, including tanespimycin [57], alvespimycin [57], retaspimycin hydrochloride (IPI-504) [57–59] [all geldanamycin analogues], ganetespib (STA-9090) [60], BIIB021 (CNF 2024) [61], luminespib (AUY922) [62], and onalespib (AT13387) [63–65]. To date, none of these agents have progressed far in clinical development, with several being discontinued due to poor tolerability.

Pimitespib (TAS 116; 3-ethyl-4-[3-(1-methylethyl)-4-[4-(1-methyl-1H-pyrazol-4-yl)-1H-imidazol-1-yl]-1H-pyrazolo[3,4-b] pyridin-1-yl] benzamide) is a selective inhibitor of HSP90α and HSP90β. Pimitespib was approved in June 2022 in Japan for the treatment of GIST that has progressed after other lines of treatment [66]. Unlike some other HSP90 inhibitors, pimitespib shows good oral availability [67], meaning it can be administered orally, thus, giving greater flexibility than intravenous-only agents.

*Administration schedule* The dose and administration schedule for pimitespib was determined from a first-in-human phase 1 study that aimed to identify the maximum tolerated dose (MTD; primary endpoint), and assess the safety, efficacy, pharmacokinetics and pharmacodynamics of pimitespib [68]. The study enrolled 61 patients in the UK and Japan (n = 60 evaluated) with histologically or cytologically confirmed advanced solid tumours (n = 7 with GIST) for which standard treatment was no longer effective. Pimitespib was administered either once daily (step 1) or every other day (QOD; step 2) in 21-day cycles. Each step comprised a dose-escalation phase to determine the MTD and a dose-expansion phase at the MTD. The MTD was 107.5 mg/m^2^ in step 1 (n = 16) and 210.7 mg/m^2^ in step 2 (n = 20). The dose-expansion phases evaluated the safety, tolerability and efficacy at the MTD of two regimens, a modified once-daily regimen (changed to 5 days on/2 days off per week, due to treatment-related adverse effects [TRAEs] requiring drug interruption seen at the MTD; n = 19) and QOD (n = 6). No correlation was found between body surface area and oral clearance and so the expansion phase doses were a flat-dose of the MTD, i.e. 160 mg/day for the modified once-daily regimen and 340 mg/day for the QOD regimen. The safety/tolerability and pharmacokinetic profiles supported the use of these dosages in subsequent studies [68].

*Anti-tumour activity* Pimitespib inhibited KIT phosphorylation and caecal GIST volume in a mouse model derived from a family with multiple GISTs caused by a germline Asp820Tyr mutation at exon 17 of *KIT* [69]. Further, pimitespib inhibited the proliferation of and induced apoptosis in imatinib-resistant and imatinib-naïve GIST cell lines [70]. In xenograft mouse models, pimitespib inhibited tumour growth in imatinib-resistant GISTs [70].

*Clinical efficacy* Pimitespib has shown good efficacy and tolerability as a fourth-line therapy in patients with GIST with no response to three previous lines of treatment in the phase 3 CHAPTER-GIST-301 trial (JapicCTI-184094) [71]. This trial enrolled 86 Japanese adults who had histologically confirmed GIST refractory to/intolerant of imatinib, sunitinib and regorafenib, and an Eastern Cooperative Oncology Group (ECOG) performance status of 0 or 1 [71]. The study consisted of a randomised, double-blind period and an open-label period. In the former, patients were randomised 2:1 to pimitespib 160 mg/day 5-days on/2-days off (n = 58) or placebo (n = 28) in 21-day cycles. In the open-label period, patients could crossover to pimitespib (if they received placebo in the double-blind period) or continue pimitespib at the same or lower dose (if they received pimitespib in the double-blind period). Blinded treatment was continued until disease progression, unacceptable toxicity, withdrawal of consent or discontinuation deemed necessary by the study investigator.

Median follow-up was 8.0 and 7.0 months for pimitespib and placebo, respectively [71]. Median PFS by blinded central radiological review (primary endpoint) with pimitespib and placebo was 2.8 versus 1.4 months, respectively (hazard ratio [HR] 0.51 [95% confidence interval (CI) 0.30–0.87]; *P* = 0.006, stratified log-rank test). Median OS according to the prespecified analysis using the rank-preserving structural failure time (a secondary efficacy endpoint) was longer with pimitespib (13.8 months [95% CI 9.2–not reached]) than with placebo (7.6 months [95% CI 5.3–14.9]) [HR 0.42; 95% CI 0.21–0.85; *P* = 0.007]. The results of the other secondary endpoints, including OS, investigator-assessed PFS, response rate, DCRs, time to progression and secondary PFS, are summarised in Table 1. Pharmacogenomic analysis (exploratory endpoint) indicated that pimitespib was more effective than placebo (PFS improvements) in patients with secondary *KIT* mutations of exons 13/14 and 17/18, as well as in the overall patient population. No significant difference was observed in health-related quality of life (HRQOL) [exploratory endpoint] between pimitespib- and placebo-treated patients [71].

**Table 1:** Secondary efficacy outcomes from the phase 3 CHAPTER-GIST-301 trial of pimitespib in Japanese patients with gastrointestinal stromal tumours [71]

	Pimitespib (N = 58)	Placebo (N = 28)	*P* value
Investigator-assessed PFS, median			
Months (95% CI)	2.9 (2.8–4.2)	1.4 (0.8–2.5)	
HR (95% CI)	0.58 (0.33–1.02)	–	0.028^a^
Secondary PFS^b^, median			
Months (95% CI)	2.7 (0.7–1.4)	NA	
Adjusted OS, median^c^			
Months (95% CI)	13.8 (9.2–NR)	7.6 (5.3–14.9)	
HR (95% CI)	0.42 (0.21–0.85)	–	0.007^a^
Unadjusted OS, median			
Months (95% CI)	13.8 (9.2–NR)	9.6 (5.5–NR)	
HR (95% CI)	0.63 (0.32–1.12)	–	0.081^a^
BCRR-assessed responses			
Complete response, n (%)	0	0	
Partial response, n (%)	0	0	
Stable disease, n (%)	36 (62.1)	10 (35.7)	
Progressive disease, n (%)	20 (34.5)	18 (64.3)	
Not assessable, n (%)	2 (3.4)	0	
DCR w/ duration ≥ 12 wk, % (95% CI)	27.6 (16.7–40.9)	21.4 (8.3–41)	0.369^d^
SD w/ duration ≥ 6 wk, % (95% CI)	62.1 (48.4–74.5)	35.7 (18.6–55.9)	0.019^d^
Time to progression, median			
Months (95% CI)	2.8 (1.6–2.9)	1.4 (0.9–1.8)	
HR (95% CI)	0.67 (0.41–1.09)	–	0.052^a^

*BCRR* blinded central radiological review, *CI* confidence interval, *DCR* disease control rate, *HR* hazard ratio, *NA* not applicable, *NR* not reached, *OS* overall survival, *PFS* progression-free survival, *SD* stable disease, *w/* with, *wk* weeks

^a^One-sided P value

^b^PFS in patients (n = 17) crossed over to pimitespib in the open-label phase

^c^Adjusted using the rank-preserving structural failure time

^d^Fisher’s exact test, one-sided

*Safety and tolerability* The tissue distribution and ocular toxicity of pimitespib were evaluated in rats, using another HSP90 inhibitor, luminespib, as a reference (given that ocular toxicity had been observed in a phase I study of luminespib [72]). In contrast to luminespib, pimitespib showed rapid distribution equilibrium between plasma and the retina, and did not accumulate in the retina [67]. Further, in contrast to luminespib, pimitespib did not cause any histological abnormalities in the retina. Evaluation of tissue distribution profiles showed marked less distribution into the retina versus tumour for pimitespib, in contrast to other HSP90 inhibitors tested (alvespimycin, ganetespib, SNX-5422, and luminespib) [67].

In CHAPTER-GIST-301 [71], adverse events (AEs) were reported by 96.6% of patients receiving pimitespib and 78.6% of those receiving placebo. The most common TRAEs (in ≥ 10% of pimitespib-treated patients) of any grade and grade ≥ 3 are summarised in Table 2. Treatment-related serious AEs were reported in six (10.3%) pimitespib recipients. Few visual abnormalities were reported in patients receiving pimitespib, with the most frequent being grade 1 night blindness (n = 8 [13.8%]); median time to resolution was 21 days and there were no associated discontinuations or dose modifications. Two cases of grade ≥ 2 abnormalities were reported (retinal vein occlusion and visual impairment), both of which resolved with discontinuation and dose interruption/reduction, respectively. The incidence of TRAEs leading to permanent discontinuation in pimitespib-treated patients was low (n = 3 [5.2%]). Further, most AEs could be managed with dose modification, rather than treatment discontinuation. There were no treatment-related deaths. These data suggest that pimitespib has an acceptable safety profile compared with existing treatments for GIST. The lack of significant difference in HRQOL between pimitespib and placebo groups suggests that the AEs associated with pimitespib did not negatively impact this parameter.

**Table 2:** Common (occurring in ≥ 10% of pimitespib recipients) treatment-related adverse events observed in the phase 3 CHAPTER-GIST-301 trial (adapted from Kurokawa et al. [71])

Treatment-related AE	Pimitespib (N = 58)		Placebo (N = 28)	
	All grades, n (%)	Grade ≥ 3, n (%)	All grades, n (%)	Grade ≥ 3, n (%)
Diarrhoea	43 (74.1)	8 (13.8)	4 (14.3)	0
Decreased appetite	18 (31.0)	1 (1.7)	2 (7.1)	0
Malaise	15 (25.9)	1 (1.7)	3 (10.7)	0
Blood creatinine increased	15 (25.9)	0	2 (7.1)	0
Nausea	14 (24.1)	0	3 (10.7)	0
Renal impairment	9 (15.5)	2 (3.4)	0	0
Night blindness	8 (13.8)	0	0	0

*AE* adverse event

#### Future directions in Japan

**Fig. 2:**
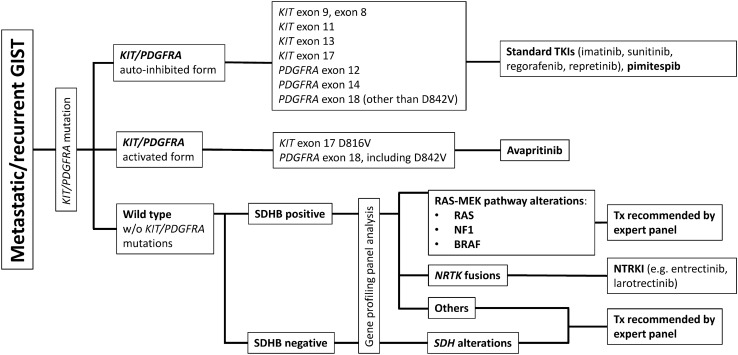
Proposed treatment pathway for metastatic/recurrent gastrointestinal stromal tumours (GIST) [3].This figure is based on Fig. 1 in Nishida et al. Cancers (Basel) 2021; 13(13): 3158 which was published under a Creative Commons Attribution CC BY 4.0 International license (https://creativecommons.org/licenses/by/4.0/); changes made were to formatting/style only. *BRAF* v-raf murine sarcoma viral oncogene homolog B1, *KIT* v-kit Hardy-Zuckerman 4 feline sarcoma viral oncogene homolog, *MEK* mitogen-activated extracellular signal-regulated kinase, *NF1* neurofibromatosis type 1, *NRTK* nonreceptor tyrosine kinase, *NTRKI* neurotrophic tyrosine kinase inhibitor, *PDGFRA* platelet-derived growth factor receptor alpha, *RAS* rat sarcoma, *SDH* succinate dehydrogenase, *SDHB* succinate dehydrogenase B subunit, *TKIs* tyrosine kinase inhibitors, *Tx* therapy, *w/o* without

Figure 2 provides a treatment pathway that illustrates how TKIs are currently used [3]. Ripretinib is currently available in a number of countries as fourth-line treatment for GIST, while avapritinib is approved for unresectable or metastatic GIST harbouring a *PDGFRA* exon 18 mutation. Neither of these drugs is approved for use in Japan. However, based on the positive results from CHAPTER-GIST-301 [71], pimitespib was approved in patients with advanced GIST refractory to imatinib, sunitinib and regorafenib in Japan in June 2022 [66].

## Upcoming treatments for advanced GIST

TKIs have revolutionised the treatment of GIST but many challenges still remain, including: treatment of patients with rarer molecular subtypes of GIST that do not respond to current TKIs (such as SDH-deficient GIST) and those who experience significant intolerance to TKIs; the need for continuous long-term TKI treatment in those who do respond (to prevent the proliferation of ‘persistent’ GIST cells); and the development of TKI resistance [73]. These limitations are driving the ongoing search for alternative approaches to the management of GIST.

### Alternative use of current TKIs as monotherapy

The possibility of using TKIs that are approved for late-line use as a strategy earlier in the course of treatment has been investigated. Ripretinib is approved in various countries in patients who have received prior treatment with ≥ 3 TKIs, including imatinib. However, the efficacy of ripretinib (vs. sunitinib) as second-line treatment was assessed in patients with advanced GIST who had progressed on or were intolerant to imatinib only. In the phase 3 INTRIGUE trial, patients were randomised to ripretinib (n = 226) or sunitinib (n = 227) [74]. Median PFS by independent radiological review (primary endpoint) was not significantly different between the two treatments, either in those with a *KIT* exon 11 primary mutation (8.3 vs. 7.0 months, respectively) or in all patients (8.0 vs. 8.3 months, respectively). The ORR was numerically higher for ripretinib than sunitinib in both patient populations; median OS was not reached in either arm. Ripretinib appeared to be better tolerated than sunitinib, with fewer grade 3–4 treatment-emergent AEs with ripretinib (41.3% vs. 65.6%, respectively).

### TKIs as combination therapy

The use of TKI-based combination therapy has also been investigated, whether with other TKIs or with agents that have alternative mechanisms of action. Bezuclastinib (CGT9486, PLX9486) is a selective type 1 inhibitor of *KIT* exon 17 and 18 mutations. A phase 1b/2a study assessed the effects of the drug in combination with sunitinib in 35 patients with refractory GIST. Median PFS was 12.1 months and the CBR was 80% [75]. Based on these encouraging results, a phase 3 study has been initiated to compare the combination of bezuclastinib plus sunitinib with sunitinib alone in patients with imatinib-resistant, sunitinib-naive GIST (NCT05208047) [76].

Binimetinib is an allosteric inhibitor of MAP kinase 1 and 2 (MEK1/2), which has been investigated in combination with imatinib for patients with advanced GIST. A phase 1b study showed only limited activity in the general cohort of patients with imatinib-resistant advanced GIST, with only one of 22 patients showing a PR and a best ORR of 4.5% [77]. A phase 2 study of the binimetinib-imatinib combination in patients with treatment-naïve advanced GIST found a confirmed PR in 29 of 42 evaluable patients, with a best ORR of 69% and a median PFS of 29.9 months [78].

The phase 1 CHAPTER-GIST-101 (NCT05245968) study is assessing pimitespib plus imatinib in patients with GIST that has progressed during or within 6 months of imatinib treatment [79]. The study consists of (1) a dose-escalation part that aims to estimate the recommended dose for further study, evaluate safety and pharmacokinetics, and observe the antitumour effect; and (2) a dose-expansion part that will evaluate the efficacy and safety of (1) pimitespib plus imatinib, (2) pimitespib monotherapy and imatinib administered after pimitespib, and (3) sunitinib monotherapy. The study has an estimated completion date of December 2023.

Selinexor, an inhibitor of chromosome region maintenance 1 protein (CRM1) [80], is being investigated in a phase 1b/2 trial in combination with imatinib for patients with imatinib-resistant unresectable and/or metastatic GIST. Preliminary results indicate good tolerability, and an ORR of 67%, with two of 12 patients (17%) achieving a PR and six patients (50%) stable disease (SD) as the best response [81]. The CBR (CR, PR, SD) at ≥ 16 weeks was 42%, and median PFS was 3.5 months [81].

Combination therapies that aim to eliminate persistent GIST cells have not yet reached clinical assessment, but an in vitro study of imatinib plus a MEK inhibitor and a phosphoinositide 3 kinase inhibitor suggests that this approach holds promise [82].

### Novel TKIs

Based on the significant role that tyrosine kinases play in GIST oncogenesis, novel TKIs are being investigated in clinical trials.

THE-630 is a next-generation TKI that has potent activity against all major classes of activating and resistance mutations observed in *KIT*-mutant GIST [83]. It is currently in phase 1/2 development in the US as second-line or greater treatment of metastatic GIST.

Dovitinib is a multi-targeted receptor TKI that was assessed in a phase 2 trial of patients with GIST who were refractory or intolerant to imatinib. DCR (CR + PR + SD) was 52.6% at 12 weeks, median PFS was 4.6 months, and median OS was not reached [84].

Another multi-targeting TKI, famitinib (a structural analogue of sunitinib), is in phase 3 development for advanced GIST after failure of imatinib (SHR1020-III-303; NCT04409223) [85, 86]. A phase 1 trial that included two patients with GIST had indicated efficacy, with one patient having achieved a PR to treatment [87].

### SDH-deficient GIST

Patients with SDH-deficient GISTs represent a difficult population to treat [88]. There have been occasional reports of successful treatment of this tumour type with TKIs, such as the VEGFR-targeted TKI pazopanib (n = 1; 16% reduction in tumour size after 17 cycles) [89]; however, in general, SDH-deficient GISTs respond poorly to TKIs [88]. In contrast, some success has been reported with temozolomide in these patients. A 100% DCR (defined as PR or stabilisation of progressing disease) was seen in five patients with SDH-deficient GIST who received temozolomide (in 4-week cycles at 85 mg/m^2^ daily for 21 days followed by 7 days off treatment) [90]. The median OS was 6.4 years from date of diagnosis and 1.9 years from the start of treatment [90]. A phase 2 study of temozolomide in advanced SDH-deficient GIST (NCT03556384) is currently underway [91].

Based on the finding that hypermethylated promoter sequences in the *SDHC* gene lead to SDH deficiency [88], the use of DNA hypomethylating agents has been investigated. A phase 2 study of the DNA methyltransferase inhibitor guadecitabine had been initiated in patients with non-*KIT/PDGFRA*-mutated GIST and SDH-deficient paragangliomas and pheochromocytomas, but enrolment has been closed due to low accrual (NCT03165721) [92, 93].

### Antibody–drug conjugates

Antibody–drug conjugates (ADCs) use a monoclonal antibody (mAb) to selectively deliver a cytotoxic drug to antigen-expressing tumour cells. GIST cells strongly express the 358-amino acid orphan G protein-coupled receptor GPR20 [94, 95]. Higher levels of expression have been observed in GIST samples that received multiple treatment lines compared with naïve/early-treated samples [96].

DS-6157a is an anti-GPR20 ADC, comprised of a mAb that targets GRP20 and an exatecan derivative topoisomerase I inhibitor [94]. DS-6157a showed cell growth-inhibitory activity in GPR20-positive GIST cells derived from a human GIST-T1 cell line [94]. A phase I study of DS-6157a in patients with advanced GIST (NCT04276415; DS6157-A-U101) [97] has been reported recently (in abstract form only). At the data cut-off, results were available for 34 patients with a median treatment duration of 9.9 weeks; only modest efficacy of DS-6157a was shown. One patient with SDH-deficient GIST had a confirmed PR, while 18 patients experienced SD and 10 had progressive disease as the best response. Median PFS across the doses tested was 3.6 months (95% CI 1.6–6.9) [98].

### Immunotherapy

Immunotherapy has become the standard of care for some tumour types, such as melanoma and non-small cell lung cancer. This approach has been investigated in GIST but with limited success when immune-checkpoint inhibitors were used as monotherapy [73]. However, the combination of an anti-programmed cell death-1 (PD-1) or anti-programmed death ligand 1 (PD-L1) antibody and imatinib was found to enhance the antitumour effects of imatinib in a genetically engineered mouse model of GIST [99]. On this basis, combinations of TKIs and immunotherapies have been investigated.

A phase 1b study found no synergistic effect of dasatinib plus ipilimumab (an anti-cytotoxic T-lymphocyte-associated antigen 4 [CTLA-4] mAb) in 28 patients with advanced GIST (refractory to or intolerant of imatinib and sunitinib) and other sarcomas [100]. No patients had PR or CR, and median PFS was 2.8 months. Nevertheless, a number of studies are underway to assess the effects of other TKI/immunotherapy combinations. Spartalizumab (PDR001; an PD-1 receptor antibody) plus imatinib is being investigated in a phase 1/2 study in patients with advanced GISTs after failure of standard TKI therapies including imatinib, sunitinib and regorafenib (NCT03609424) [101].

A phase 2 trial is ongoing to assess the efficacy and safety of the combination of the TKI axitinib and the anti-PD-L1 antibody avelumab in patients with unresectable/metastatic GIST who have received no more than 3 lines of treatment, including imatinib and sunitinib (NCT04258956) [102].

A further phase 1/2 study is assessing the effects of avelumab and regorafenib in patients with advanced or metastatic solid tumours, including GIST (NCT03475953) [103].

Immunotherapy combinations are also being investigated, including several studies of ipilimumab plus nivolumab (a mAb targeting PD-1/PDCD-1) [104]. To date, results have been reported from only one trial [105]. In this phase 2 study of patients with advanced/metastatic GIST who had failed to respond to or were intolerant of at least imatinib (median of 3 prior lines of therapy), patients were randomised to nivolumab alone or nivolumab plus ipilimumab. The combination did not appear to provide significant benefit compared with nivolumab alone; there were no objective responses in the nivolumab arm (10/19 patients had SD; CBR 52.6%; median PFS 11.7 weeks), and one of 16 patients had a CR in the combination arm (4/16 had SD; CBR 31.3%; median PFS 8.3 weeks).

## Conclusion

In Japan and internationally, treatments have been established for GIST that has not responded to up to two lines of treatment. In many countries, ripretinib can be used as a fourth-line treatment and avapritinib has been approved for *PDGFRA* exon 18-mutated GIST, while pimitespib is now approved as fourth-line treatment for GIST in Japan. Although it may be expected that the establishment of pimitespib in the treatment armamentarium will improve patient prognosis in Japan, there are still very limited options for patients with this stage of disease. As such, the development of new drugs and combination therapies remains an important goal.

## Data Availability

Data sharing is not applicable to this article as no data sets were generated or analyzed during its development.
